# Gewichtsdiskriminierung

**DOI:** 10.1007/s11577-021-00799-z

**Published:** 2021-09-27

**Authors:** Friedrich Schorb

**Affiliations:** Institut für Public Health und Pflegeforschung, Universität Bremen, Grazer Straße 2, 28259 Bremen, Deutschland

## *Strings, Sabrina*:

Fearing The Black Body. The Racial Origins of Fat Phobia. New York: New York University Press. 284 Seiten. ISBN 978-1-4798-8675‑3. Preis: $ 28,–.

Im Sommer 2020 wurden die USA von einer doppelten Krise gebeutelt. Rassistische Polizeigewalt provozierte die größten Proteste seit den 1960er-Jahren. Gleichzeitig geriet das Coronavirus völlig außer Kontrolle. Genau wie von der Polizeigewalt waren auch von der Pandemie Afroamerikanerinnen und Afroamerikaner überdurchschnittlich oft betroffen. Doch während infolge des Mordes an George Floyd das Thema Rassismus bei Polizei und Justiz große Aufmerksamkeit erhielt, wurde die häufigere Betroffenheit von Afroamerikanerinnen und Afroamerikanern durch COVID-19 in der Öffentlichkeit kaum diskutiert. Dort, wo man schließlich doch nach Gründen für die erhöhte Sterblichkeit innerhalb der afroamerikanischen Bevölkerung suchte, wurde Adipositas als vermeintliche Ursache gefunden. Die Soziologin Sabrina Strings widersprach dieser Wahrnehmung am 25. Mai 2020 in einem Gastbeitrag für die New York Times mit dem Titel „It’s not Obesity, it’s Slavery“. Nicht Adipositas also, sondern Sklaverei, deren Erbe das Leben der afroamerikanischen Bevölkerung bis heute in vielfacher Weise präge, seien in letzter Konsequenz auch für die vielen Corona-Todesfälle innerhalb der afroamerikanischen Community verantwortlich, argumentierte Strings. Zum Erbe des Sklavensystems zählte sie dabei die schlechte soziale Lage, die geringen finanziellen Ressourcen, die prekären Wohn- und Arbeitsbedingungen und den eingeschränkten Zugang zum Gesundheitswesen als Teile eben jener strukturellen Diskriminierungen, die auch für die vielen Polizeiübergriffe gegenüber Afroamerikanerinnen und Afroamerikanern verantwortlich seien.

Zum Erbe von Sklaverei und weißer Vorherrschaft gehört für Strings auch die hegemoniale Missachtung dicker schwarzer Körperlichkeit. Den rassistischen Ursprüngen der dominanten Körperfettphobie forscht sie in ihrem 2019 erschienen Buch „Fearing the Black Body. The Racial Origins of Fat Phobia“ nach. Sie tut dies, indem sie die vielfach erzählte Geschichte westlicher Schönheitsideale mit der Darstellung und Deutung schwarzer Körper durch weiße Kunstschaffende, Schriftstellerinnen und Schriftsteller, Journalistinnen und Journalisten sowie Wissenschaftlerinnen und Wissenschaftlern kombiniert.

Strings beginnt ihre Ausführungen mit den Venusdarstellungen von Albrecht Dürer und Paul Peter Rubens und kontrastiert diese mit den Bildern schwarzer Sklavinnen aus derselben Epoche, die als dürre kränkliche und unscheinbare Schatten ihrer opulenten Herrinnen porträtiert werden. Doch dieses Bild der gleichermaßen blassen wie üppigen Venusdamen und ihrer schlanken schwarzen Dienerinnen verändert sich Strings zufolge im späten 17. Jahrhundert als Dünnsein zunehmend als Ausdruck insbesondere männlicher Rationalität wahrgenommen wird. Dem schlanken, gepflegten Idealbild des rationalen Mannes wird jetzt das Bild des dicken, dummen und lebensfrohen Tollpatsches und Raufboldes gegenübergestellt, etwa in der Figur des Falstaffs von Shakespeare.

Passend dazu definieren zur selben Zeit auch frühe Vertreter eines „wissenschaftlichen Rassismus“ neben Hautfarbe und Schädelformen noch weitere Merkmale wie Wohlbeleibtheit als rassische Charakteristika und verknüpften sie mit Charaktereigenschaften wie Gefräßigkeit, Faulheit und Promiskuität. Ganz im Sinne der damals populären Klimatheorie unterstellten sie, dass den Afrikanerinnen und Afrikanern die süßen Früchte des Landes ganzjährig ohne große Anstrengung zur Verfügung stünden und sie sich daher unbeschwert der Völlerei und der Wollust hingeben könnten. Diese traumhaften Zustände aber verhinderten die Reifung des Menschen bzw. der Männer zu vernunftbegabten, rationalen, beherrschten und damit auch schlanken Wesen, die in der Lage seien, sich die Erde untertan zu machen.

Dem imaginierten Paradies wurde durch die Vertreter der weißen Vorherrschaft mit der „Verwandlung von Afrika in ein Geheg zur Handelsjagd auf Schwarzhäute“ (Karl Marx) ganz real ein brutales Ende gesetzt. Ironischerweise führten nun aber ausgerechnet die süßen Früchte des transatlantischen Sklavenhandels in Form von Rohrzucker dazu, dass sich die europäische Medizin verstärkt mit ernährungsbedingten Krankheiten auseinanderzusetzen begann und damit auch der Debatte um die medizinischen Folgen von Körperfett Auftrieb gab.

Den Höhepunkt der Kolonialzeit begleiteten sogenannte Völkerschauen, in denen Bewohnerinnen und Bewohner der kolonialisierten Gebiete in Menschenzoos einem Massenpublikum vorgeführt wurden. Als Vorgänger dieses Phänomens können die Auftritte der Khoikhoi Sarah Baartman zu Beginn des 19. Jahrhunderts gelten, die dem begeisterten europäischen Publikum als „Hottentot Venus“ präsentiert wurde. Ihre körperlichen Rundungen, allen voran ihr Fettsteiß, wurden zum Symbol sexuell aufgeladener, rassistischer Überlegenheitsfantasien.

In radikaler Abkehr von wohlbeleibten weiblichen Schönheitsvorstellungen setzte sich zum Ende des 19. Jahrhunderts in US-amerikanischen Frauenzeitschriften der Mittel- und Oberschicht ein ultraschlankes Schönheitsideal durch. Die hier präsentierten schmalen, hochgewachsenen und sportlichen Frauenkörper waren immer auch blond und nord- oder westeuropäischer Provenienz. Den Neueingewanderten aus Irland, später aus Süditalien und den jüdischen Schtetln Osteuropas hingegen wurde das Weißsein und die damit einhergehende genetische und ästhetische Überlegenheit abgesprochen. Wo die Hautfarbe nicht zur Verfügung stand, mussten andere körperliche Unterschiede konstruiert werden, denen zufolge diese Völker Afrikanerinnen und Afrikanern ähnlicher seien als den Abkömmlingen von Angelsachsen und Germanen. Und zu diesen Distinktionskriterien, zeigt Strings, wurde immer auch ihre vermeintliche Dickleibigkeit gezählt.

Von den ästhetischen Idealen in Malerei und Mode wechselt Strings im letzten Abschnitt ihres Buches zur medizinischen Beschäftigung mit einem hohen Körpergewicht: Repräsentiert wird diese Debatte im Buch in der Person von John Harvey Kellogg. Kellogg, heute bekannt als Erfinder der Cornflakes, war zu Lebzeiten weniger am Aufbau eines globalen Lebensmittelkonzerns interessiert als an der Etablierung eines Sanatoriums zur Behandlung chronischer Krankheiten. Kellogg empört sich gleichermaßen über Fettleibigkeit wie über den aus seiner Sicht übertriebenen Schlankheitswahn weißer US-amerikanischer Frauen, da sie deren Fruchtbarkeit und Fortpflanzungsfähigkeit in Frage stelle. Wie andere Eugeniker seiner Zeit war Kellogg lediglich am Wohlergehen der „weißen Rasse“ interessiert und ohnehin fest davon überzeugt, dass People of Colour aufgrund ihrer genetischen Unterlegenheit langfristig vom Aussterben bedroht seien.

An diesem Punkt zieht Sabrina Strings den Zusammenhang zur Gegenwart, indem die niedrigere Lebenserwartung der afroamerikanischen im Vergleich zur weißen Bevölkerung ebenfalls als Zeichen einer nicht systembedingten, sondern selbstverschuldeten gesundheitlichen Unterlegenheit gewertet wird.

Strings Arbeit liest sich flüssig und überzeugt durch gelungenes „academic story telling“. Der Spannungsbogen über die Jahrhunderte hinweg bleibt dabei zwangsläufig ausschnitthaft. Vor allem die medizingeschichtlichen Rückblicke sind mit der ausschließlichen Konzentration auf die Person John Harvey Kelloggs zu kurz geraten. Die Frage, ob die universalen auf dem Body Mass Index basierenden Grenzwerte die afrikanische und afroamerikanische Bevölkerung überproportional pathologisieren und damit benachteiligen, wird zwar angesprochen, aber nicht zu Ende diskutiert. Hier bieten sich Ansatzpunkte für weitere Forschungsarbeiten.

Strings deutet die Abwertung schwarzer Körper als minderwertig, krank und fettleibig als Teil einer inhärent rassistischen Biopolitik auf allen gesellschaftlichen Ebenen. „Fearing the Black Body“ fügt damit der Debatte um die strukturellen Ursachen von Gewichtsdiskriminierung, die bislang stark über die Auseinandersetzung mit Sexismen dominiert war, eine wichtige intersektionale Ergänzung hinzu. Das Buch dürfte daher schon bald ein Standardwerk der Fat Studies werden.

